# Multi-Omics Unravels Metabolic Alterations in the Ileal Mucosa of Neonatal Piglets Receiving Total Parenteral Nutrition

**DOI:** 10.3390/metabo13040555

**Published:** 2023-04-13

**Authors:** Junkai Yan, Yuling Zhao, Lu Jiang, Ying Wang, Wei Cai

**Affiliations:** 1 Division of Pediatric Gastroenterology and Nutrition, Xinhua Hospital, School of Medicine, Shanghai Jiao Tong University, Shanghai 200092, China; 2 Shanghai Institute for Pediatric Research, Shanghai 200092, China; 3 Shanghai Key Laboratory of Pediatric Gastroenterology and Nutrition, Shanghai 200092, China; 4 Department of Pediatric Surgery, Xinhua Hospital, School of Medicine, Shanghai Jiao Tong University, Shanghai 200092, China

**Keywords:** total parenteral nutrition, fatty acyl-carnitines, succinate, fatty acid oxidation, citrate cycle

## Abstract

Total parenteral nutrition (TPN) is life-saving therapy for the pediatric patients with intestinal failure (IF) who cannot tolerate enteral nutrition (EN). However, TPN-induced metabolic alterations are also a critical issue for the maintenance of intestinal homeostasis, and thus the global metabolomic signatures need to be addressed. In this study, ileal mucosal biopsies were collected from 12 neonatal Bama piglets receiving either EN or TPN for 14 days, and changes in the intestinal metabolism were examined by multi-omics (HM350 Metabolomics + Tandem Mass Tag (TMT)-based proteomics). As a result, a total of 240 compounds were identified by metabolomics, including 56 down-regulated and 9 up-regulated metabolites. Notably, tissue levels of fatty acyl-carnitines (decreased by 35–85%) and succinate (decreased by 89%) dramatically decreased in the TPN group, suggestive of disrupted processes of fatty acid oxidation (FAO) and the citrate cycle, respectively. Interestingly, however, no differences were found in the production of adenosine 5′-triphosphate (ATP) between groups, suggesting that these dysregulated metabolites may have mainly led to the loss of bioactive compounds rather than energy deficit. Additionally, 4813 proteins were identified by proteomics in total, including 179 down-regulated and 329 up-regulated proteins. The analysis of protein–protein interactions (PPI) indicated that most of the differentially expressed proteins were clustered into “lipid metabolism” and “innate immune responses”. In summary, this work provided new findings in TPN-induced intestinal metabolic alterations, which would be useful to the improvement of nutritional management for IF patients.

## 1. Introduction

For decades, nutritional support including parenteral nutrition (PN) and enteral nutrition (EN) has been a gold standard practice in treatment for pediatric patients with intestinal failure (IF), as well as preterm neonates lacking sufficient enteral feeding due to immature gastrointestinal development [1,2,3]. In particular, total parenteral nutrition (TPN) has been considered a life-saving therapy for those patients who cannot tolerate EN, including patients with severe short bowel syndrome (SBS) and chronic intestinal pseudo-obstruction (CIPO). On the other hand, however, increasing evidence has also suggested that TPN may serve as a “double-edged sword” as it may cause significant intestinal injury, villus atrophy, barrier dysfunction, impaired mucosal immunity and disrupted entero-hepatic circulation, leading to severe complications such as parenteral nutrition-associated liver disease (PNALD) which represents 16–60% of total death in long-term PN [4]. We assume that metabolic alterations may be implicated in TPN-related intestinal injury, and thus a systemic examination using multi-omic approaches is needed for a better understanding of the global metabolic signatures.

Proteomic profiling is a large-scale comprehensive investigation of proteins, including information on protein abundance and their interacting networks, while metabolomics profiling is a comprehensive biochemical technique to assess systemic metabolism, reflecting the “net effects” of genetic, transcriptomic, proteomic and environmental interactions [5,6]. By using metabolomic approaches, numerous studies on PN, including both animal models and clinical trials, have demonstrated that dysregulated gut microbial or bile acid profiles [7,8,9] may contribute to the development of TPN-related intestinal injury and subsequent PNALD through multiple pathways (e.g., farnesoid X receptor signaling). By using metabolomic approaches, one of our previous works has also raised a new possibility that TPN may induce significant changes in the expression of intestinal drug metabolism-related genes, which may in turn affect the pharmacokinetic process of certain drugs, in particular glutathione-related detoxication and ATP-binding cassette (ABC) transporter-dependent elimination [10]. However, as the intestine is one of the most important digestive organs with multiple functions, including nutrient digestion and absorption, microorganism defense, immune response and hormone secretion, integrated metabolic networks are therefore crucial for a holistic analysis of functional changes under TPN. It would remain elusive how metabolic changes under TPN can systemically influence the network of these intestinal functions unless a global metabolomic signature could be identified with multi-omics. Therefore, this study aimed to depict a landscape of TPN-induced intestinal metabolic changes by integrating metabolomics and proteomics, which may be imperative for the understanding of TPN-related intestinal dysfunction as well as for the translation of potentially useful metabolites in clinical practice.

## 2. Materials and Methods

### 2.1. TPN Piglet Model

Twelve 2-day-old Guangxi Bama minipigs (0.7–1 kg) were housed in a light-controlled room with a 12 h light/dark cycle, randomized to the EN and TPN groups (*n* = 6/group). Energy intake and composition of macronutrients of the two groups were basically identical as described previously [11]. EN piglets were fed a cow milk-based formula at 48.6 g/kg/d suspended in 240 mL water, providing 197 kcal energy by 25 g/kg/d/lactose, 12.5 g/kg/d protein and 5 g/kg/d fat, composed of 40% medium chain triglycerides (MCT) and 60% long chain triglycerides (LCT). For the TPN group, piglets were parenterally fed with TPN solution containing 240 mL/kg/day (240 mL fluid, 197 kcal energy) of protein (13 g), lipid (5 g) and carbohydrate (25 g). The detailed TPN formula is shown in Table 1. The surgery procedure has been described previously elsewhere [12]. All animals were sacrificed after 14 days of TPN infusion. Ileal mucosal biopsies were isolated about 10 cm proximal to the ileocecal valve. All the samples were stored at −80 °C until further use.

### 2.2. Intestinal Morphology Assessment

Formalin-fixed and paraffin-embedded tissue sections (5 μm) were stained using standard immunohistochemistry procedures. Villus height and crypt depth were measured in 20+ well oriented, full-length crypt/villus units per specimen. Data were analyzed with Leica LAS AF LITE image processing software 2.6.0 (Leica, Mannheim, Germany).

### 2.3. Metabolomic Profiles and Data Processing

Metabolomic profiles (*n* = 6/group) were identified with a commercially available kit (High Throughput Targeted Quantification Kit for Metabolites, HM350 Metabolome, BGI, Shenzhen, China). Briefly, 100 μL of tissue lysates were mixed with 100 μL of a 50% water/methanol solution containing an internal standard mix as quality control. After centrifugation at 18,000× *g* 4 °C for 10 min, 100 μL of supernatant was taken for LC-MS/MS analysis on QTRAP 6500+ (SCIEX, Framingham, MA, USA). Chromatographic separation was performed on a BEH C18 (2.1 mm × 10 cm, 1.7 μm, waters) with the temperature maintained at 40 °C. Raw data were processed with Compound Discoverer 3.1 (Thermo Fisher Scientific, Waltham, MA, USA), and the resulting metabolomic profiles were identified through BGI Library. Differential metabolites were defined according to the following criteria: log2 fold change (FC) > 1 or <−1, adjusted *p* value < 0.05. Bioinformatics analyses were then performed, including Kyoto Encylopaedia of Genes and Genomes (KEGG) pathway classification (https://www.kegg.jp, accessed on12 July 2021).

### 2.4. Proteomic Profiles and Data Processing

Protein extracts (*n* = 3/group) were generated by homogenizing 20 mg tissue in 200 μL lysis buffer (4% Sodium dodecyl sulfate (SDS), 100 mM Dithiothreitol (DTT), 150 mM Tris-HCl pH 8.0). Procedures for Tandem mass tag (TMT)-labelling and proteome analysis using LC-MS/MS were performed as described previously elsewhere [10]. Briefly, TMT-labelled peptides were loaded onto a C18-reversed phase column in buffer A (2% acetonitrile and 0.1% formic acid) and separated with a linear gradient of buffer B (90% acetonitrile and 0.1% formic acid) at a flow rate of 300 nL/min over 60 min. The dynamic exclusion duration was 30s. Survey scans were acquired at a resolution of 70,000 at *m*/*z* 200, and the resolution for the high-energy collisional dissociations (HCD) spectra was set to 17,500 at *m*/*z* 200. The normalized collision energy was 30 eV. The resulting raw files were imported into MaxQuant software (version 1.6.0.16, Max Planck Institute of Biochemistry, Martinsried, Germany) for data interpretation and protein identification. Differentially expressed proteins were defined according to the following criteria: unique peptides ≥ 1, fold change > 1.3 or <0.7, adjusted *p* value < 0.05. Bioinformatics analyses were then performed, including KEGG pathway classification (https://www.kegg.jp, accessed on 12 July 2021)), Gene Ontology (GO)-term classification (david.abcc.ncifcrf.gov, accessed on 1 December 2021) and protein–protein interaction (PPI) networks (v10, string-db.org (accessed on 12 August 2021)).

### 2.5. Biochemical Measurement

Total levels of adenosine 5′-triphosphate (ATP) were determined using ATP Quantification Kit (Cat no. S0026, Beyotime, Shanghai, China). Acetyl-CoA content was determined using Acetyl-CoA Content Assay Kit (Cat no. ml077327, mlbio, Shanghai, China).

### 2.6. Statistical Analysis

In metabolomic profiling and proteomic profiling, adjusted *p* < 0.05 was used for the screening of differential metabolites and proteins followed by bioinformatics analysis. For simple comparisons, data were statistically analyzed and plotted using GraphPad Prism 8.0 (GraphPad Software Inc., San Diego, CA, USA). All results are presented as mean ± SD. Variables were analyzed by Student’s t-test, and a difference is considered significant when *p* < 0.05.

## 3. Results

### 3.1. General Status of TPN Piglets

No differences were found in body weight between groups. Morphologically, however, 14d-TPN administration induced significant intestinal villus atrophy, as characterized by decreased villus height (256.33 vs. 191.76 μm, *p* < 0.01) and crypt depth (123.41 vs. 83.77 μm, *p* < 0.01) (Table 2).

### 3.2. Metabolomic Profiles Altered by TPN

**Figure 1:**
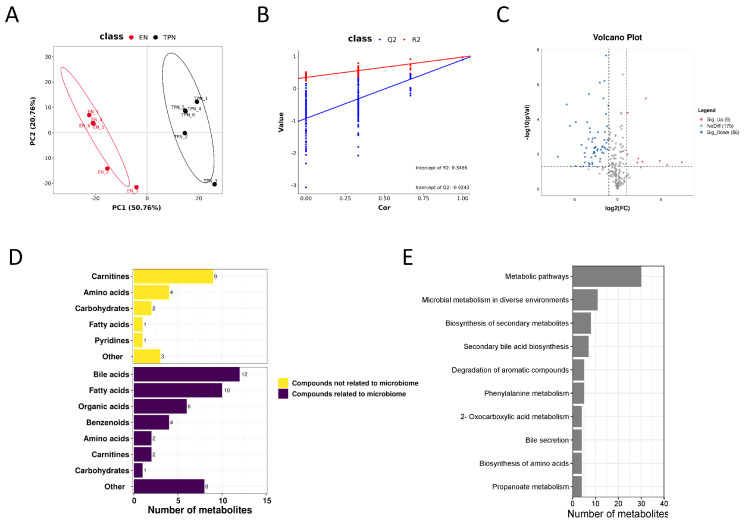
Metabolomic profiles in the ileum epithelium. (**A**,**B**) Partial Least Squares Discriminant Analysis (PLSDA) and validation plots from the enteral nutrition (EN) and total parenteral nutrition (TPN) piglets. (**C**) Volcano plots showing differential metabolites. (**D**) Classification of identified differential metabolites. (**E**) Kyoto Encylopaedia of Genes and Genomes (KEGG) pathway enrichment analysis of differential metabolites.

**Figure 2:**
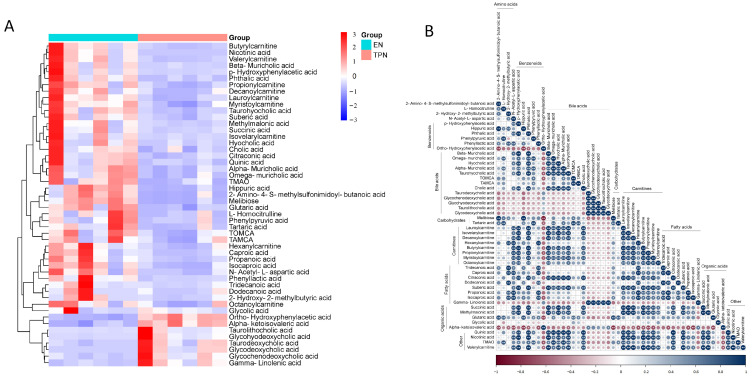
Top 50 differential metabolites between EN and TPN. (**A**) Hierarchical clustering of top 50 differential metabolites. (**B**) Correlation matrix of top 50 differential metabolites. Color indicates the value of correlation coefficient (* *p* < 0.05, ** *p* < 0.01).

In total, 240 compounds with identification information were determined, and the partial least squares-discriminant analysis (PLS-DA) showed obvious separation of the identified metabolites between groups (Figure 1A,B). Among all the metabolites, 56 down-regulated and 9 up-regulated metabolites were identified as differential metabolites, as indicated in volcano plots (Figure 1C). Among the 65 differential metabolites, 45 metabolites were related to the microbiome, including bile acids (12), fatty acids (10), organic acids (6), benzenoids (4), amino acids (2), carnitines (2), carbohydrates (1) and others (8); 20 were not related to the microbiome, including carnitines (9), amino acids (4), carbohydrates (2), fatty acids (1), pyridines (1) and others (3) (Figure 1D). KEGG pathway enrichment analysis revealed that most of the differential metabolites were clustered into “metabolic pathways”, “microbial metabolism in diverse environments” and “biosynthesis of secondary metabolites” (Figure 1E). The top 50 differential metabolites were shown in the heatmap (Figure 2A), and their correlations were displayed in a correlation matrix plot (Figure 2B).

### 3.3. Proteomic Profiles Altered by TPN

**Figure 3:**
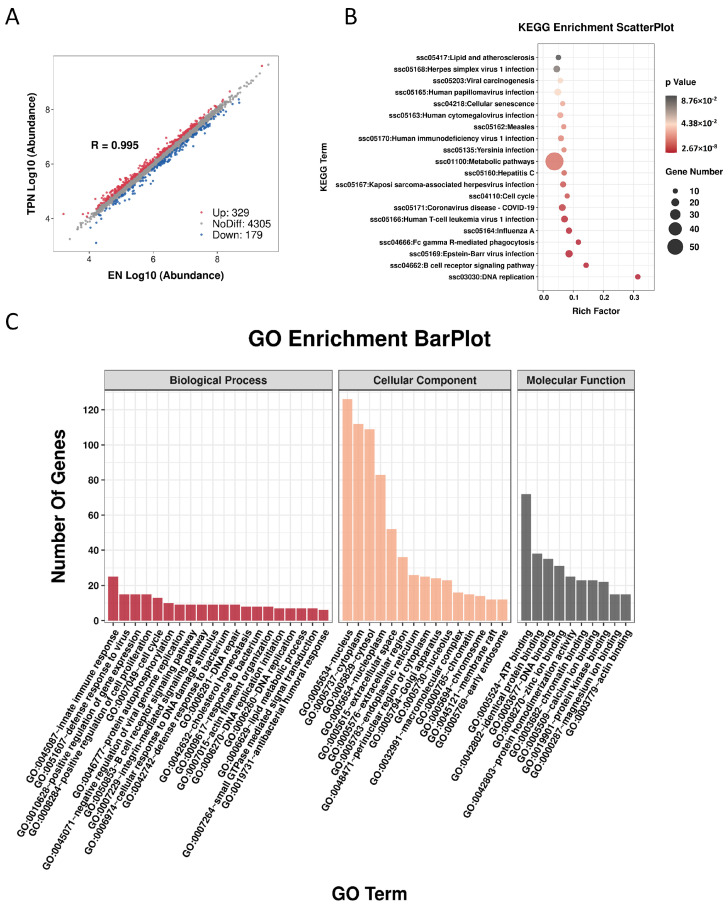
Proteomic profile in the ileum epithelium. (**A**) Scatter plotting of differential proteins. (**B**) KEGG pathway enrichment analysis of differential proteins. (**C**) Gene Ontology (GO) enrichment analysis of differential proteins.

**Figure 4:**
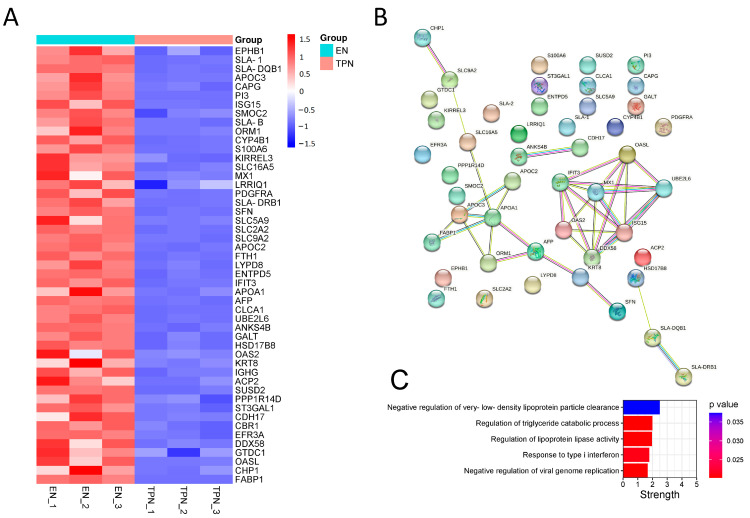
Top 50 down-regulated proteins in TPN piglets. (**A**) Heatmap of top 50 down-regulated proteins in TPN piglets. (**B**) Protein–protein interaction (PPI) analysis among top 50 down-regulated proteins. (**C**) Enrichment strength evaluation of PPI.

A total of 4813 proteins were identified, including 179 down-regulated and 329 up-regulated proteins (Figure 3A). The KEGG scatter plot indicated that most of the differential proteins were enriched in “metabolic pathway” (Figure 3B). In the GO-term enrichment analysis, the top 20 cluster annotations of Biological Process (BP) were shown, including “lipid metabolic process” (Figure 3C). The top 50 down-regulated proteins were shown in the heatmap (Figure 4A). Of note, protein–protein interaction (PPI) analysis suggested two groups: (a) FABP1/APOA1/APOC2/APOC3 involved in the regulation of lipid catabolic process, and (b) MX1/OAS2/IFIT3/ISG15/DDX58/OASL/UBE2L6 involved in the regulation of immune responses (Figure 4B). The top five interaction strengths are shown in Figure 4C. On the other hand, the top 50 up-regulated proteins were shown in Figure S1A, along with the PPI analysis shown in Figure S1B.

### 3.4. Disrupted Fatty Acid Oxidation (FAO) by TPN

**Figure 5:**
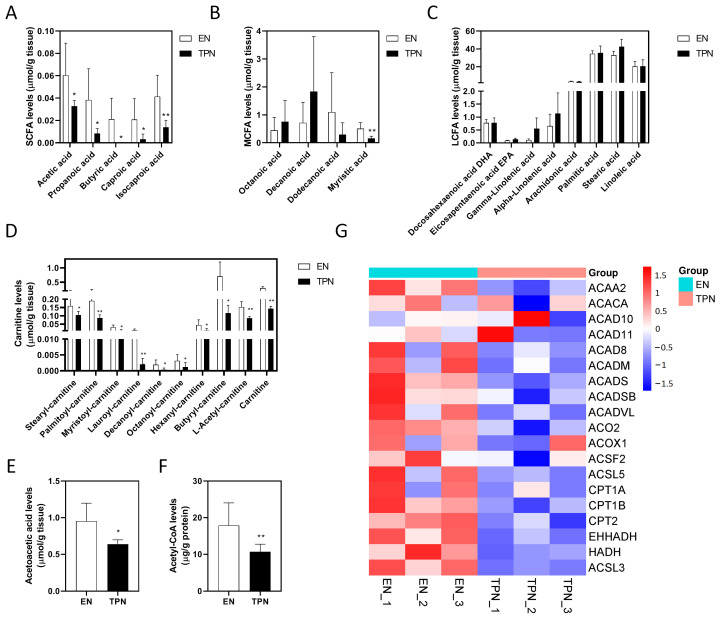
Changes in the metabolomic and proteomic profiles of fatty acid oxidation (FAO). (**A**) Tissue levels of short chain fatty acids (SCFA). (**B**) Tissue levels of medium chain fatty acids (MCFA). (**C**) Tissue levels of long chain fatty acids (LCFA). (**D**) Tissue levels of fatty acylcarnitines. (**E**) Tissue levels of acetoacetic acid. (**F**) Tissue levels of acetyl-CoA. Data are presented as mean ± SD. * *p* < 0.05, ** *p* < 0.01 (*n* = 5–6/group). (**G**) Heatmap of proteins involved in FAO.

As shown, the tissue levels of short chain fatty acids (SCFAs) decreased significantly in the TPN group, including acetic acid (decreased by 46%), propanoic acid (decreased by 78%), butyric acid (undetectable in TPN), caproic acid (decreased by 85%) and isocaproic acid (decreased by 66%) (Figure 5A). Among the medium chain fatty acids (MCFAs), only myristic acid decreased significantly in the TPN group (decreased by 71%) (Figure 5B). However, no significant differences were found in the long chain fatty acids (LCFAs) between groups (Figure 5C). Of note, all of the fatty acyl-carnitines decreased significantly in the TPN group, including stearyl-carnitine (decreased by 35%), palmitoyl-carnitine (decreased by 55%), myristoyl-carnitine (decreased by 68%), lauroyl-carnitine (decreased by 85%), decanoyl-carnitine (decreased by 82%), octanoyl-carnitine (decreased by 63%), hexanoyl-carnitine (decreased by 73%), butyryl-carnitine (decreased by 84%), L-acetyl-carnitine (decreased by 45%) and carnitine (decreased by 52%) (Figure 5D). As the end products of FAO, acetoacetic acid levels and acetyl-CoA levels in the TPN group decreased by approximately 34% and 42%, respectively (Figure 5E,F). Consistently, a number of proteins involved in mitochondrial and peroxisomal FAO were evidently down-regulated by TPN (Figure 5G). An overview of the disrupted FAO process was plotted in KEGG map “fatty acid degradation” (Figure S2). Taken together, these results suggested that SCFA deficiency and the disruption of the intestinal FAO process was evident under TPN.

### 3.5. Disrupted Citrate Cycle by TPN

**Figure 6:**
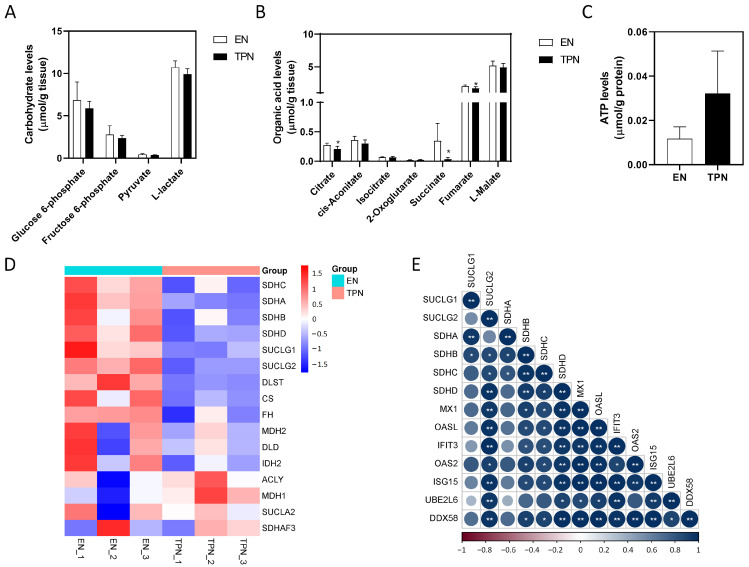
Changes in the metabolomic and proteomic profile of the citrate cycle. (**A**) Tissue levels of carbohydrates involved in glycolysis. (**B**) Tissue levels of organic acids involved in TCA cycle. (**C**) Tissue levels of Adenosine 5′-triphosphate (ATP). Data are presented as mean ± SD. * *p* < 0.05 (*n* = 5–6/group). (**D**) Heatmap of proteins involved in TCA cycle. (**E**) Correlation matrix of proteins involved in succinate metabolism and anti-viral immune responses. Color indicates the value of correlation coefficient (* *p* < 0.05, ** *p* < 0.01).

For the main products of glycolysis, no significant differences were found in glucose 6-phosphate, fructose 6-phosphate, pyruvate or L-lactate (Figure 6A). For the main intermediates of the citrate cycle, tissue levels of citrate (decreased by 25%, *p* < 0.05) and fumarate (decreased by 19%, *p* < 0.05) slightly decreased in the TPN group, and notably succinate decreased by 89% (*p* < 0.05) in the TPN group (Figure 6B). However, no significant changes were found in adenosine 5′-triphosphate (ATP) production between the groups (Figure 6C). Consistently, key enzymes involved in the metabolism of succinate evidently were down-regulated by TPN, including succinate dehydrogenases (SDHs) and succinate-CoA ligases (SUCLGs) (Figure 6D). An overview of disrupted Citrate cycle was plotted in the KEGG map “citrate cycle (TCA cycle)” (Figure S3). In order to address the potential impact of succinate deficiency, we investigated the correlation of proteins involved in succinate metabolism and immune responses. As shown, the abundance of SUCLG2, SDHB, SDHC and SDHD was significantly correlated with MX1/OAS2/IFIT3/ISG15/DDX58/OASL/UBE2L6, which was a group of interacting proteins involved in immune responses as described above (Figure 6E). Taken together, these data suggested that some of the main intermediates of the citrate cycle were dysregulated by TPN. ATP production was not affected, however, and the immune responses may be repressed due to succinate deficiency.

## 4. Discussion

Application of PN in pediatrics has increased rapidly over the last 20 years. In 2019, 389 children (prevalence of 30 per million children) received PN in the UK, a number that has almost doubled since 2012 [13]. In France, 385 patients on home PN aged 0–19 years were recorded (prevalence of 2.6 patients/100,000 inhabitants), with an incidence of about 50–60 new cases per year [14]. In the past 50 years, PN has become a standard-of-care, lifesaving clinical support therapy for premature infants as well as patients who cannot tolerate EN, and particularly TPN has been the only approach of nutritional support for those patients with severe intestinal failure. However, despite the significant clinical benefit of PN, evidence is also accumulating that exclusive PN or TPN may lead to increasing associated risks of exacerbating intestinal injury, due in part to reduced gastrointestinal blood flow and the disruption of intestinal homeostasis, including villus atrophy [15,16], decline in mucosal immunity [17,18] and disassembly of tight junctions leading to hyperpermeability [19,20]. By using rodents as a laboratory TPN model, a variety of aberrantly activated signaling pathways have been identified, including PI3K/pAkt [21], TLR4/EGF [22] and NF-κB/MLCK [23]. However, given the physiological differences between rodents and humankind that cannot be neglected (e.g., the anatomy of the gastrointestinal tract, omnivorous diet, milk composition, body composition and litter bearing), the extent to which these experimental findings with laboratory rodents can represent human infants and pediatric patients remains controversial. Instead, from a comparative biology perspective, the piglet model has more anatomical, physiological, immunological and metabolic similarities with humans than any other rodent model [24]. Therefore, the piglet model has become a well-recognized model in emerging areas of human nutrition and gut microbiota, development and host immune function, as well as the pathology and therapeutic approaches for the treatment of complex human diseases that originate during infancy [25].

In this study, we performed proteomics and metabolomics (multi-omics) in neonatal piglets in order to depict the whole picture of metabolic changes within intestinal mucosa as a result of TPN. Our previous work has reported significant changes in intestinal drug metabolism by TPN [10], but changes in the metabolism of other nutrients (e.g., carbohydrates, fatty acids, amino acids, bile acids etc.) remain to be addressed. Unlike previous studies using either fecal or serum samples [12,26], this study investigated the intestinal metabolic changes by directly using ileal mucosal tissues, because changes in the metabolites within epithelium in situ may have been largely influenced by the microbiome if fecal samples were used. There are several key findings from this work. First, substantial alterations in metabolites and metabolic proteins were identified in TPN piglets. Most of them were down-regulated, suggestive of compromised diversity of metabolites. Second, down-regulated proteins were mainly enriched in two clusters, lipid metabolism and innate immune responses. Third, impairment of the FAO process and the citrate cycle in TPN was evident.

In this study, we found that all the indicated SCFAs decreased significantly in the TPN group. These findings were rational because one of our previous works has demonstrated that the abundance of SCFA-producing *Lactobacillus* decreased dramatically in the ileal content of TPN piglets [27]. In children, studies using fecal samples have also demonstrated that the abundance of SCFAs (butanoic and pentanoic acids) in SBS patients weaning on PN was significantly lower than their counterparts weaning of PN [28,29], concomitant with reduced abundance of SCFA-producing bacteria such as *Lachnospiraceae* and *Ruminococcaceae*. To date, intestinal SCFAs have been shown to play multiple roles in the maintenance of intestinal homeostasis, including serving as an energy source for enterocytes and a modulator of the gut barrier [30]. In this study, as the PPI profile suggested two main clusters of protein interactions, lipid metabolism and innate immune responses, we focused on discussing the role of SCFAs in the regulation of gut immunity. Overall, SCFAs may contribute to the regulation of host health or disease via two main mechanisms. One is the regulation of target cell epigenetics by the inhibition of histone deacetylases (HDACs). For instance, the effect of butyrate to protect against gut inflammation has been proven to be dependent on the inhibition of HDACs in macrophages and dendritic cells to down-regulate pro-inflammatory cytokines, e.g., IL-1b, IL-6 and IL-8 [31]. Additionally, the other is the activation of G protein-coupled receptors (GPRs) expressed in immune cells. A recent study has reported that IL-22 production was elevated by the activation of GPR41 via SCFAs, thereby protecting against inflammation and promoting gut homeostasis [32]. Unlike SCFAs, however, most of the MCFAs and LCFAs remained unchanged in TPN, which is partially inconsistent with previous findings. By using jejunal tissues from a mouse model of TPN, Feng et al. found that the content of LCFAs were dramatically changed, including α-linolenic acid (decreased by 57%), eicosapentaenoic acid (decreased by 61%) and arachidonic acid (increased by 63%) [33]. These differences might be attributed to either the sampling from a different bowel segment (jejunum vs. ileum) or different animal species of modeling (mice vs. piglets). As it is known, mitochondrial FAO is an important pathway for maintaining energy homeostasis, especially during fasting when glucose and glycogen stores are low. Typically, free fatty acids need to be esterified with carnitine in the cytoplasm before being transported into mitochondria, a key reaction catalyzed by carnitine-palmitoyl transferases (CPT1/2). In this study, the content of MCFAs and LCFAs remained unchanged, although fatty acyl-carnitines were found to be substantially decreased in the TPN group, suggesting that this key step for FAO was evidently impaired. Given that recent works have demonstrated that FAO may play a major role in the maintenance of intestinal stem cells and renewal of the epithelium [34,35], we hypothesize that suppressed FAO may serve as a potentially novel therapeutic target for treating TPN-induced villus atrophy, and thus further studies are required to address this possibility.

In addition to the suppressed FAO process, another one of the key findings in this study is the disruption of the citrate cycle, an important biological process in energy metabolism and providing intermediates for the synthesis of some amino acids. By using urine samples, Nuria et al. reported significantly decreased concentrations of citrate and succinate in critically ill preterm newborns receiving PN, suggesting a dysregulated citrate cycle and energy deficit [36]. Likewise, in this study we found succinate decreased dramatically in the TPN group along with citrate and fumarate, suggestive of an impaired citrate cycle. Interestingly, however, the content of ATP remained unchanged, suggesting that energy supply within enterocytes was not affected by TPN. One possibility is that the ATP consumption in intestinal mucosa might have been reduced as well due to intestinal dysmotility under TPN, in spite of decreased ATP production. Instead, reduced succinate may have affected other biological processes than energy supply. For instance, succinate is produced as an intermediate metabolite formed from the conversion of succinyl-CoA, and is then oxidized by succinate dehydrogenase to form fumarate, which has been shown to be of importance in the classical activation of macrophages [37]. Additionally, the accumulation of cytosolic succinate can induce protein succinylation and consequent stabilization of the transcription factor hypoxia-inducible factor-1α [38]. Moreover, succinate/succinate receptor 1 (SUCNR1) signaling in intestinal Tuft cells has emerged as a mechanism for monitoring microbial metabolism in the intestinal lumen, leading to Tuft and Goblet cell expansion, and to an increase in type 2 cytokine production, including IL-13 [39,40].

A strength of our study is that though previous studies have reported impaired intestinal homeostasis under TPN, this present study is the first one looking into the global metabolic changes using multi-omics. However, there are two limitations of this study which must be mentioned. First, this study was limited by the small sample size of neonatal TPN piglets. Second, we did not look into mitochondrial function (e.g., mitochondrial potential or mitochondrial ATP production), because fresh samples instead of frozen samples were required. As a result, it remains unclear if mitochondrial function was affected by TPN, despite the fact that the mitochondrial FAO process along with the citrate cycle was substantially impaired.

## 5. Conclusions

In this study, we identified unique intestinal metabolic changes in a piglet model of TPN. For proteomic changes, differentially expressed proteins were enriched in lipid metabolism and innate immune responses. For metabolomic changes, decreased concentrations of fatty acyl-carnitines suggesting a disrupted FAO process, and decreased concentrations of citrate cycle intermediates (e.g., succinate) potentially contributing to immune-regulation, were of importance under TPN.

## Figures and Tables

**Table 1:** Components of TPN solution.

Component	Volume (mL)
50% dextrose	50
8.5% amino acids	153
20% SOLE	25
10% sodium chloride	2.4
10% potassium chloride	2.4
Trace elements mix	0.5
Water-soluble vitamins mix	0.5
Fat-soluble vitamins mix	0.5
10% calcium gluconate	4.6
Sodium glycerophophate	4.6
magnesium sulfate	0.33
Total	243.83

SOLE: soybean oil-derived lipid emulsion (Lipofundin^®^, B. Braun Melsungen, AG, Melsungen, Germany).

**Table 2:** General status of TPN piglets.

Indicators	EN	TPN
Body weight (onset, kg)	1.09 ± 0.24	0.92 ± 0.21
Body weight (sacrifice, kg)	2.36 ± 0.12	1.59 ± 0.47
Villus height (μm)	256.33 ± 45.01	191.76 ± 28.56 **
Crypt depth (μm)	123.41 ± 29.38	83.77 ± 27.69 **

** *p* < 0.01.

## Data Availability

The data presented in this study are available on request from the corresponding author. The data are not publicly available due to privacy.

## Supplementary Materials

The following supporting information can be downloaded at: https://www.mdpi.com/article/10.3390/metabo13040555/s1, Figure S1: Top 50 up-regulated proteins in TPN piglets; Figure S2: KEGG pathway plotting for the altered proteins and metabolites involved in fatty acid degradation; Figure S3: KEGG pathway plotting for the altered proteins and metabolites involved in Citrate cycle.
