# Supplementary material for: Multi-Omics Unravels Metabolic Alterations in the Ileal Mucosa of Neonatal Piglets Receiving Total Parenteral Nutrition
Source: Metabolites. 2023 Apr 13;13(4):555. doi: 10.3390/metabo13040555 (PMC10144288; doi:10.3390/metabo13040555)
Supplement: Supplementary file 1 [file metabolites-13-00555-s001.zip › metabolites-2297527-supplementary.pdf]

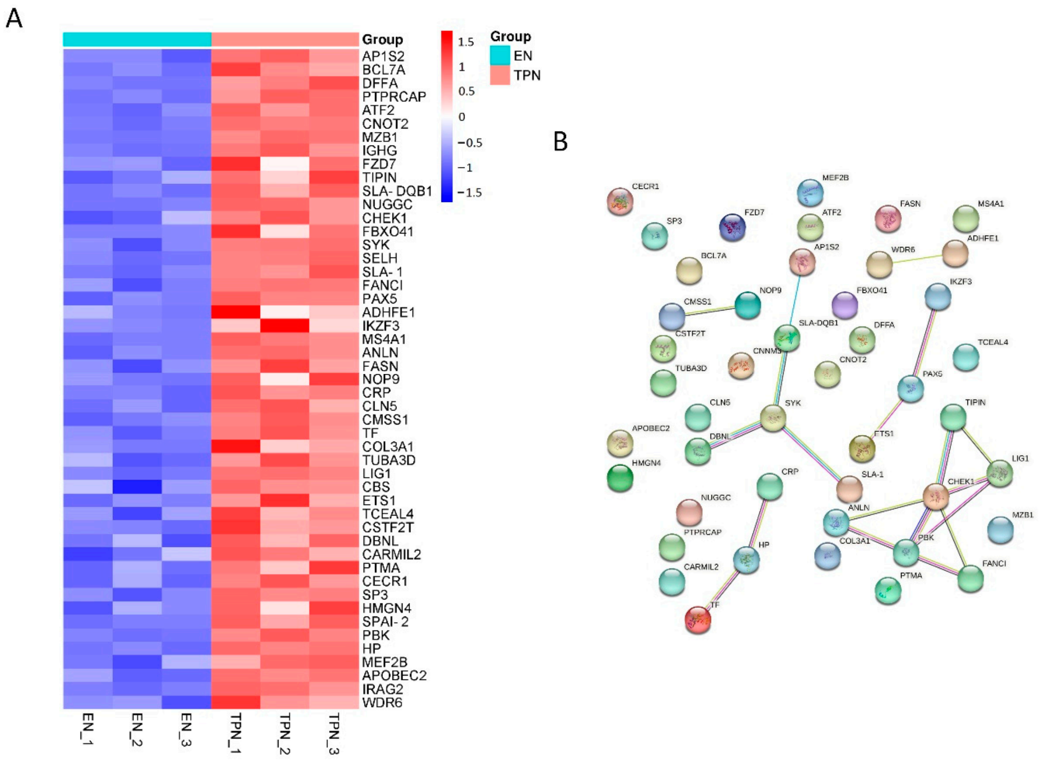

Figure S1.Top 50 up-regulated proteins in TPN piglets (A) Heatmap of top 50 up-regulated proteins in TPN piglets. (B) Protein-protein interaction (PPI) analysis among top 50 up-regulated proteins.

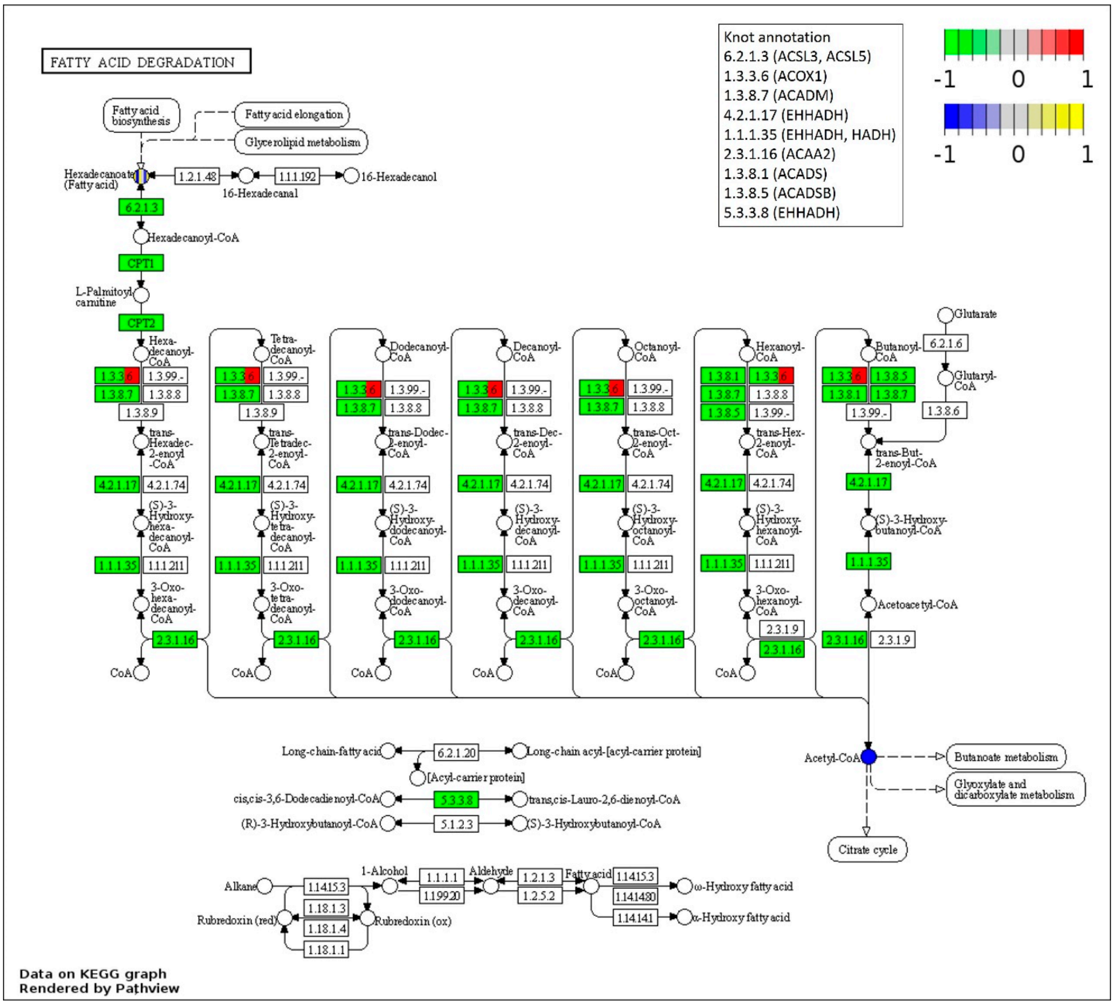

Figure S2. KEGG pathway plotting for the altered proteins and metabolites involved in fatty acid degradation.

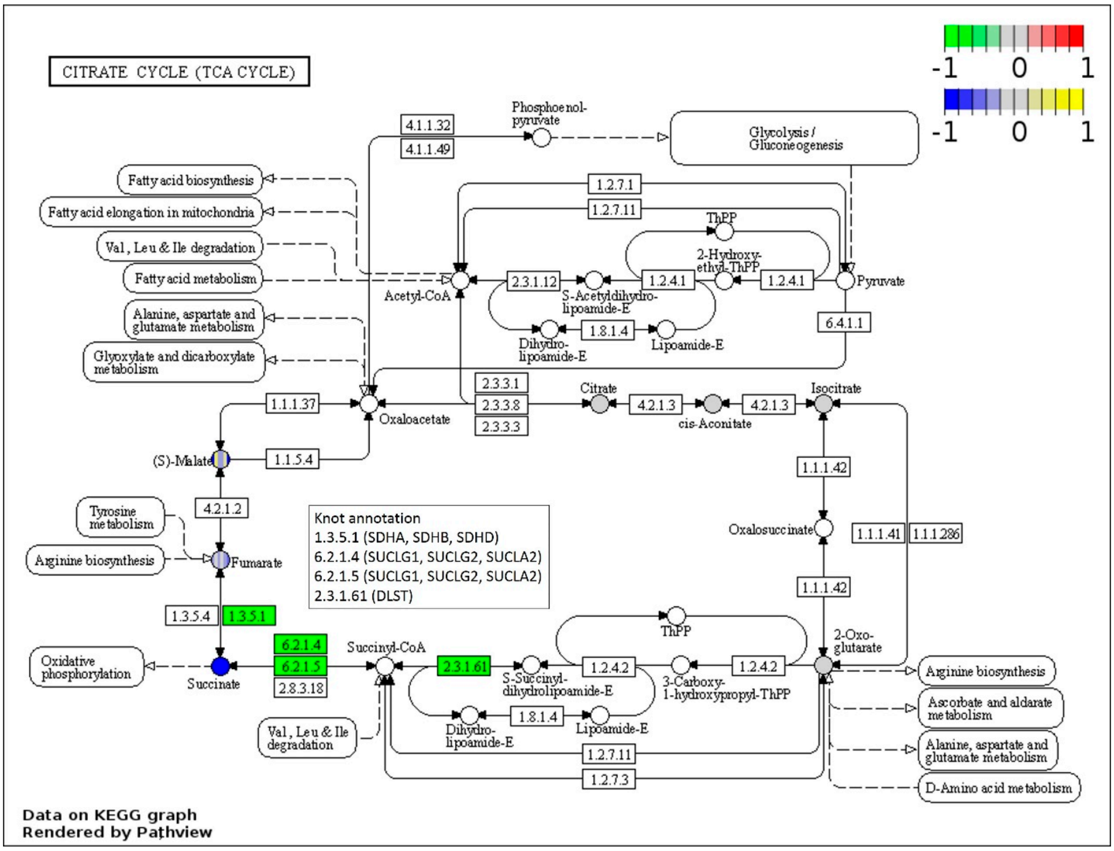

Figure S3. KEGG pathway plotting for the altered proteins and metabolites involved in Citrate cycle.
